# Rv3033, as an Emerging Anti-apoptosis Factor, Facilitates Mycobacteria Survival via Inhibiting Macrophage Intrinsic Apoptosis

**DOI:** 10.3389/fimmu.2018.02136

**Published:** 2018-09-21

**Authors:** Wei Zhang, Qian Lu, Yuanshu Dong, Yan Yue, Sidong Xiong

**Affiliations:** Jiangsu Key Laboratory of Infection and Immunity, Institutes of Biology and Medical Sciences, Soochow University, Suzhou, China

**Keywords:** mycobacteria, Rv3033, macrophages, apoptosis, intrinsic pathway

## Abstract

Apoptosis inhibition is a critical strategy of mycobacteria facilitating its survival in macrophages, but the underlying mechanism is not completely understood. In this study, we found that Rv3033, a secreted virulence factor of mycobacteria, played an important role in bacillary survival within macrophages. Forced over-expressed of Rv3033 in macrophages could efficiently resist mycobacteria-induced early and late apoptosis, accompanied with the obvious increased cellular bacterial burden. By exploring the underlying mechanism, we found that Rv3033 efficiently repressed the intrinsic (caspase-9 meditated), but not the extrinsic (caspase-8 mediated) apoptotic pathway in mycobacteria-infected macrophages. And this repression relied on the orchestrating blockade of both mitochondrial cytochrome c release and endoplasmic reticulum (ER) stress PERK branch activation. Our study uncovered a novel function of mycobacterial virulence factor Rv3033 as an anti-apoptotic protein, which may provide a new target for tuberculosis (TB) treatment.

## Introduction

Tuberculosis is an infectious disease caused by the intracellular pathogen mycobacteria, which results in 1.7 million deaths and 6.3 million new cases annually worldwide (1). Mycobacteria infection has become increasingly severe due to the increasingly mobile population, the emergence of drug-resistant mycobacteria strains, and the epidemic of co-infection with HIV (2).

As the first line of host defense, macrophages are key immune cells in resistance against mycobacteria (3–5). Long-term struggling with macrophages, mycobacteria has developed various immune modulation strategies to facilitate its survival (6). Among them, inhibiting apoptosis is one of the major mechanisms (7–9). For example, virulence factors, such as SodA, NuoG, Eis, and Rv3364c, have been found to potently inhibit host cell apoptosis by blocking either the TNF-α-mediated pathway or the mitochondria pathway (10–14), and these virulence factors represent the possible targets for future prophylactic and therapeutic regimes. Therefore, identification of new virulence factors that could potently alter macrophage apoptosis has becoming a hot spot in the field of anti-tuberculosis work.

In 2005, Rengarajan and colleagues screened a genome-wide mycobacteria transposon mutant library and established several candidate factors that are essential for mycobacteria survival in macrophages (15). Based on their work, we screened four secreted proteins, Rv0928, Rv1096, Rv3033, and Rv3369, and assessed their effects on macrophage apoptosis. Interestingly, we found that Rv3033 had a robust inhibitory effect on macrophage apoptosis. Herein, we tried to elucidate Rv3033 anti-apoptotic effect and the corresponding mechanism in the context of mycobacteria infection.

In this study, we found that Rv3033 had higher expression in the virulent strain H37Rv than the avirulent strain BCG and H37Ra, could suppress mycobacteria-induced macrophage apoptosis and enhance the bacillary survival. In addition, this inhibitory effect relied on the repressing intrinsic apoptosis by orchestrating blockade of mitochondrial cytochrome c release and endoplasmic reticulum (ER) stress PERK branch activation. In conclusion, this study revealed the anti-apoptosis role of Rv3033 in mycobacteria-infected macrophages, and modulating Rv3033 expression might represent a new therapeutic target for controlling TB.

## Materials and methods

### Cells, mice, and bacteria culture

RAW264.7 and HEK293T cells (ATCC) were cultured in Dulbecco's modified Eagle's medium (DMEM, Sigma-Aldrich) supplemented with 10% fetal bovine serum (FBS), 2 mM L-glutamine, and 1% penicillin- streptomycin. Cells were cultured in a humidified incubator at 37°C and 5% CO_2_. Mice were purchased from the Experimental Animal Center of the Chinese Academy of Sciences (Shanghai, China).All animal-related experimental procedures were performed in accordance with the guidelines for the Care and Use of Laboratory Animals (Ministry of Health, China, 1998). The guidelines were approved by the Ethics Committee of Soochow University. BMDMs were prepared as previously described (16). Briefly, femurs and tibias from C57B6/C mice were dissected, and bone marrow were flushed out. The isolated cells were filtered through a nylon mesh and plated in RPMI 1640 containing M-CSF, 10% FBS, 2 mM L-glutamine, 1% penicillin- streptomycin for 6 days. The purity of BMDMs was >90% as determined by FACS analysis using FITC-conjugated anti-F4/80 monoclonal antibody (BD Biosciences) and PE-conjugated anti-CD11b monoclonal antibody (BD Biosciences).The *M. tuberculosis* strains BCG (ATCC35733), H37Ra and H37Rv were grown in Middlebrook 7H9 broth medium supplemented with 10% ADC, 0.5% glycerol, and 0.05%Tween-80 at 37°C. M. smegmatis mc^2^155 (Ms), Ms::Vector, Ms::Rv0928, Ms::Rv1096, Ms::Rv3033, and Ms::Rv3369 were grown in Luria-Bertani medium supplemented with 0.05% Tween-80.

### BCG, H37Ra, and H37Rv pellets isolation

For mycobacterial pellets isolation, BCG, H37Ra, and H37Rv were first grown in 7H9 medium to mid-log phase and then sub-cultured into the 7H9 media at a starting OD600 of 0.1. Cultures were harvested when they reached an OD600 of 1.0 and were centrifugation at 8200^*^g for 15 min to generate pellets isolation, then were denatured by heating for 30 min at 100°C.

### Cloning and expression of Rv0928, Rv1096, Rv3033, and Rv3369

The construction of recombinant strains was performed using Ms. The Rv0928, Rv1096, Rv3033 and Rv3369 genes (Tuberculist database) were cloned into the pMV261 vector in frame with a C-terminal flag-tag. All constructed gene were electroporated into Ms. The selected pMV261 (MS::Vector) and pMV261-Rv0928, Rv1096, Rv3033, and Rv3369 (Ms::Rv0928, Ms::Rv1096, Ms::Rv3033, and Ms::Rv3369) were cultured in Luria-Bertani medium supplemented with 0.05%Tween-80 containing 50 μg/ml kanamycin. These recombinant strains were identified by western blot using an anti-flag antibody. The following primers were used:

Ms::Rv0928 Forward: 5′-CCGGAATTC TTGAAACTCAACCGATTTGGTGC

Ms::Rv0928 Reverse: 5′-CAGAAGCTTTCACTTGTCGTCATCGTCTTTGTAGTCG GCGATCG CGTTGAC

Ms::Rv1096 Forward: 5′-CGCGGATCCGTGCCGAAGCGACCCGAC

Ms::Rv1096 Reverse: 5′-CCCAAGCTTTTACTTGTCGTCATCGTCTTTGTAGTCCATCGCACC GTTATTTGGCCC

Ms::Rv3033 Forward: 5′-CCGGAATTC ATGGCTCACTCGATCGTTCG

Ms::Rv3033 Reverse: 5′-CAGAAGCTTCTACTTGTCGTCATCGTCTTTGTAGTCCTCGGGGTG GTCATCGA

Ms::Rv3369 Forward: 5′- CCGGAATTCATGTGGGCAGGCTACCGTTG

Ms::Rv3369 Reverse: 5′-CAGAAGCTTTCACTTGTCGTCATCGTCTTTGTAGTCGCCCGTGGG CGTC.

### Retroviral vector construction and retrovirus packaging

The Rv3033 gene (Tuberculist database) was amplified by polymerase chain reaction (PCR) using H37Rv genomic DNA as a template. The PCR primers (pMSCV-eGFP-Rv3033Forward:5′-CCGCTCGAGATGGATTACAAGGATGACGACGATAAGGCTCACTCGATCGTTCGCACG Reverse: 5′-AAGGTTAACCTACTCGGGGTGGTCAT CGA) contained XhoI and HpaI restriction enzyme sites. The PCR products were digested and ligated into a pMSCV-eGFP retroviral vector to form pMSCV-eGFP-Rv3033. HEK293T cells were cultured in 10 cm dishes at 37°C and 5% CO_2_ in a humidified atmosphere. After reaching 70–80% confluency, 293T cells were co-transfected with the retroviral expression vector pMSCV-eGFP-Rv3033 and packaging vector pcl-Ampho using Lipofectamine 3000 (Invitrogen). Culture supernatants were harvested at 48 and 72 h, filtered with a 0.45-μm pore size filter and concentrated with ultracentrifugation at 144000^*^g for 2 H. The viruses were resuspended in PBS and stored at −80°C.

### pMSCV-eGFP-Rv3033 RAW264.7 stable cell line construction

RAW264.7 cells were infected with viral supernatants collected from HEK293T cells transfected with retroviral constructs. After 72 h, GFP-positive cells were sorted by flow cytometry, and Rv3033 expression was verified by western blot analysis. RAW264.7 cells infected by pMSCV-eGFP and pMSCV-eGFP-Rv3033 viruses were named RAW-Vector and RAW-Rv3033 cells, respectively. Then the viability of RAW-Vector and RAW-Rv3033 cells were detected by CCK8 assay.

### BCG and H37Rv CFU assays

The CFU assay was performed as previously described (15). Briefly, RAW-Vector and RAW-Rv3033 cells were seeded into 6-well plates. Adherent monolayers were infected with BCG or H37Rv (MOI = 10). After 8 h, monolayers were changed to DMEM medium containing 200 μg/ml of amikacin to kill extracellular bacteria. On day 2, macrophages were lysed with 1 ml water plus 0.05% Triton X-100. Then, 50 μl lysates were added to 7H10 plates and cultured for 3 weeks to count colony-forming units.

### Apoptosis analysis by flow cytometry

RAW264.7 cells were seeded into 6-well plates, and adherent monolayers were infected with Ms::Vector, Ms::Rv0928, Ms:: Rv1096, Ms:: Rv3033, or Ms:: Rv3369 (MOI = 10) for 48 h. The apoptotic cells were measured by FACS using an Annexin V/PI kit (eBioscience, San Diego, CA, USA). BMDM cells were seeded into 6-well plates, and adherent monolayers were infected with Ms::Vector or Ms:: Rv3033 (MOI = 10) for 48 h. The apoptotic cells were measured by FACS using an Annexin V/PI kit (eBioscience, San Diego, CA, USA). RAW-Vector and RAW-Rv3033 cells were seeded into 6-well plates, and adherent monolayers were infected with BCG (MOI = 10) with or without pretreatment for 1 h with TUDCA (500 μg/ml) for 36 h. The apoptotic cells were measured by FACS using Annexin V-7-AAD kit (eBioscience, San Diego, CA, USA). RAW-Vector and RAW-Rv3033 cells were seeded into 6-well plates, and adherent monolayers were infected with H37Ra for 36 h. The apoptotic cells were measured by FACS using Annexin V/7-AAD kit (eBioscience, San Diego, CA, USA).

### TUNEL assay

RAW264.7 cells were seeded into 6-well plates, and adherent monolayers were infected with BCG or H37Rv (MOI = 10) for 24 h. TUNEL staining was performed using an *in situ* Cell Death Detection Kit, TMR red (Roche). Briefly, cells were washed three times with PBS, fixed with 4% paraformaldehyde for 1 h and permeabilized with 0.1% Triton-X-100 for 2 min on ice. TdT enzyme was used for BrdUTP incorporation into DNA nicks at 37°C for 1 h. The cells were washed twice with PBS and incubated with DAPI for 15 min at room temperature. Cells were washed again, analyzed using a fluorescence microscope and counted.

### Isolation of mitochondria and cytoplasm

RAW-Vector and RAW-Rv3033 cells were infected with BCG at an MOI of 10 for 24 h. Whole cell lysates and mitochondria were obtained with a cell mitochondria isolation kit according to the manufacturer's protocol (Beyotime, China).

### Western blot

Cells were harvested and lysed in lysis buffer for 30 min on ice. Equal amounts of protein from each sample were separated by SDS–PAGE and electro-blotted onto PVDF membranes. The membrane was blocked with 5% dry milk for 2 H and incubated with primary antibodies overnight at 4°C. The membranes were then incubated with relevant secondary antibodies at room temperature for 1 h and visualized using ECL (Thermo Pierce, Rockford, Illinois, USA) according to the manufacturer's instructions. Bands were quantified by ImageJ image software using β-tubulin as internal controls. The primary antibodies used in the present study were anti-caspase-3, anti-caspase-8, anti-caspase-9, anti-caspase-12, anti-Bip, anti-Chop, anti–phospho (Ser-51)-eIF2α, anti-cytochrome c, anti-Bax, anti-β-tubulin (Cell Signaling), anti-Rv3033.

### NGS and transcriptional analysis

RAW-Vector and RAW-Rv3033 cells were seeded into 6-well plates, and adherent monolayers were infected with BCG (MOI = 10) for 12 h. Total RNA was extracted by TRIzol reagent. NGS was sequencing by Genewiz.

### Statistical analysis

The data are represented as the mean ± SEM and were analyzed using GraphPad Prism software. One-way ANOVA followed by Turkey's *post hoc* test or two-way ANOVA was used for statistical analysis. ^*^ *p* < 0.05; ^**^ *p* < 0.01; ^***^ *p* < 0.001.

## Results

### Different apoptosis levels and consequent distinct bacillary loads were evidenced in H37Rv- and BCG-infected macrophages

**Figure 1:**
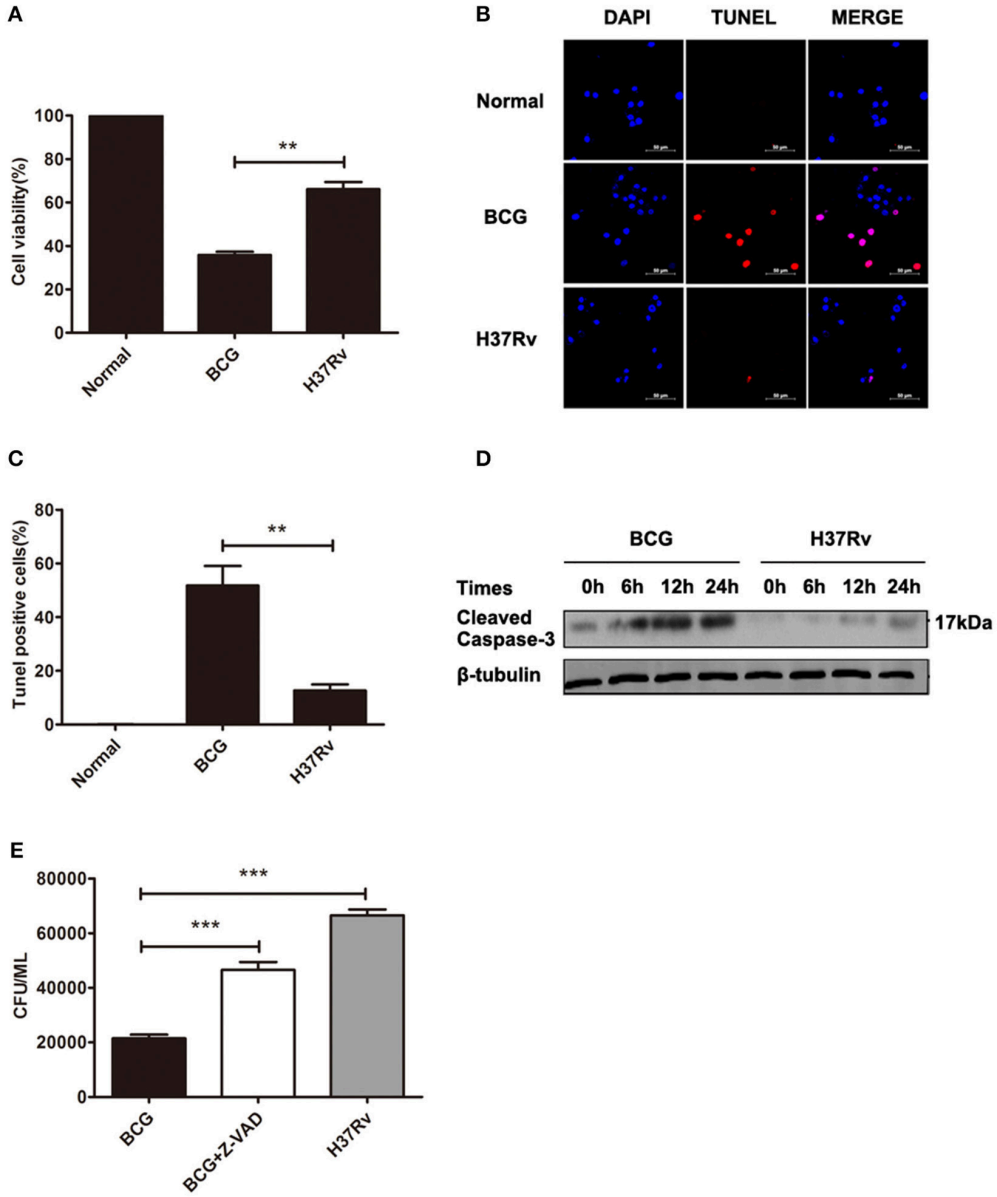
Different levels of apoptosis were induced by H37Rv and BCG infections, which led to distinct bacillary loads in macrophages. **(A)** RAW264.7 cells were infected with H37Rv or BCG (MOI = 10) for 24 h, and cell viability was determined by CCK8 assays. **(B)** DNA fragmentation was observed by TUNEL assays. The scale bars indicate 50 μm. **(C)** The percentages of apoptosis in TUNEL assays. **(D)** RAW264.7 cells were infected with H37Rv or BCG (MOI = 10) for 6, 12, and 24 h, and cleaved caspase-3 was measured by western blot. **(E)** RAW264.7 cells were infected with H37Rv, BCG, or BCG (MOI = 10) pretreated for 1 h with Z-VAD (20 μM) for 8 h, and CFU counting of intracellular viable bacteria was performed for 2 days. Data are performed as the mean (±SEM) and from one representative experiment of three independent experiments. (^**^p < 0.01; ^***^p < 0.001).

Macrophage line RAW264.7 cells were infected with distinct virulent mycobacteria (virulent H37Rv or avirulent BCG) for 24 h, and cell death was evaluated by CCK8, TUNEL, and western blot assays. As shown in Figures 1A–D, fewer apoptosis occurred in H37Rv-infected macrophages compared with the counterpart infected with BCG, reflected by the lower apoptotic cell percentage (50 vs. 15%) and active caspase-3 level, and this limited apoptosis led to the better bacillary replication in H37Rv-infected macrophages, as a much higher bacillary load was observed in H37Rv-infected macrophages compared with the counterpart infected with BCG. Meanwhile, the bacillary survival was also significantly increased in ZVAD-pretreated group, due to the obviously decreased macrophage apoptosis (Figure 1E).

### Rv3033 potently suppressed macrophage apoptosis induced by mycobacteria infection

**Figure 2:**
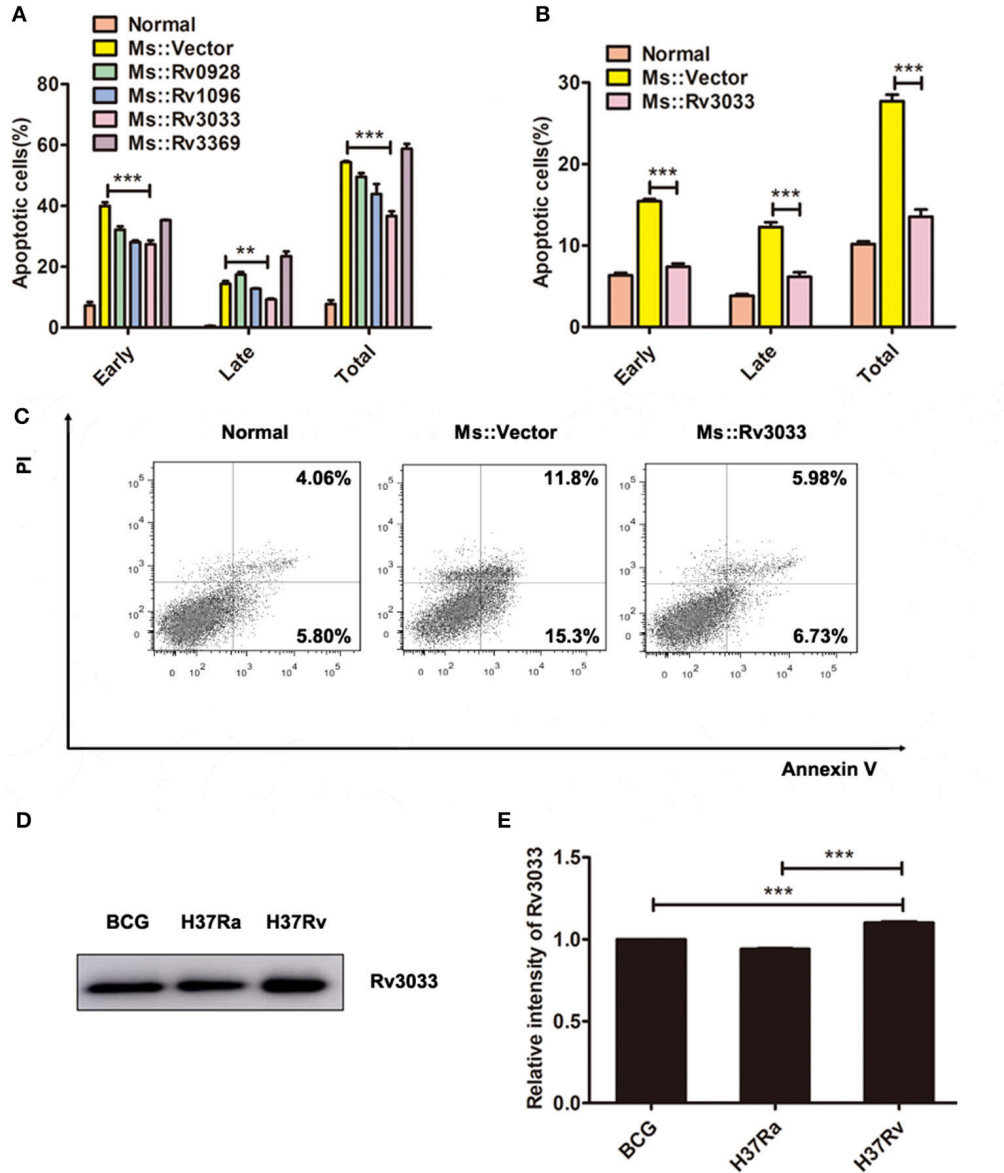
Rv3033 potently suppressed mycobacteria-induced macrophage apoptosis. **(A)** RAW264.7 cells were infected with Ms::Vector, Ms::Rv0928, Ms::Rv1096, Ms::Rv3033, or Ms::Rv3369 (MOI = 10) for 48 h. The percentages of early, late, and total apoptotic cells in FACS assay. **(B,C)** BMDM cells were infected with Ms::Vector or Ms::Rv3033 for 48 h, apoptotic cells were determined by Annexin V/PI staining assays. The percentages of early, late and total apoptotic cells in FACS assay. **(D)** The protein expression levels of Rv3033 in BCG, H37Ra, and H37Rv pellets were determined by western blot. **(E)** Semi-quantification of Rv3033. Data are performed as the mean (±SEM) and from one representative experiment of three independent experiments. (^**^p < 0.01; ^***^p < 0.001).

Mycobacterial virulence factors play significant roles in promoting mycobacteria survival in macrophages (11, 12, 17–23). Rengarajan et al. (15) identified several genes that support mycobacteria survival in macrophages by screening a genome-wide mycobacteria transposon mutant library. Based on their work, here we focused on four secreted factors, Rv0928, Rv1096, Rv3033 and Rv3369, and tried to explore their influence on macrophage apoptosis. By overexpressing these four genes in *M. smegmatis* individually, we found that compared with other recombinant bacteria, Rv3033 possessed the most potential anti-apoptotic function, evidenced by the lowest macrophage apoptosis level post *M. smegmatis* infection (Figure 2A). We also evaluated the apoptotic ration of primary murine macrophages (bone marrow-derived macrophages, BMDMs) post Rv3033-expressing Ms (Ms:: Rv3033) infection. Compared with the control group (Ms::Vector-infected BMDMs), Ms::Rv3033-infected BMDMs showed much lower total (AnnexinV^+^,27%vs13%), early (AnnexinV^+^/PI^−^, 15%vs7%) as well as late (AnnexinV^+^/PI^+^,12%vs6%) apoptotic rations (Figures 2B,C), indicating the obviously decreased apoptosis. Consistently, we found that Rv3033 expression level in BCG was comparable with that in H37Ra, both of them were much lower than that in H37Rv (Figures 2D,E), further suggesting the anti-apoptosis role of Rv3033 in mycobacteria-infected macrophages.

### Rv3033 decreased mycobacteria-infected macrophage apoptosis and promoted bacillary survival

**Figure 3:**
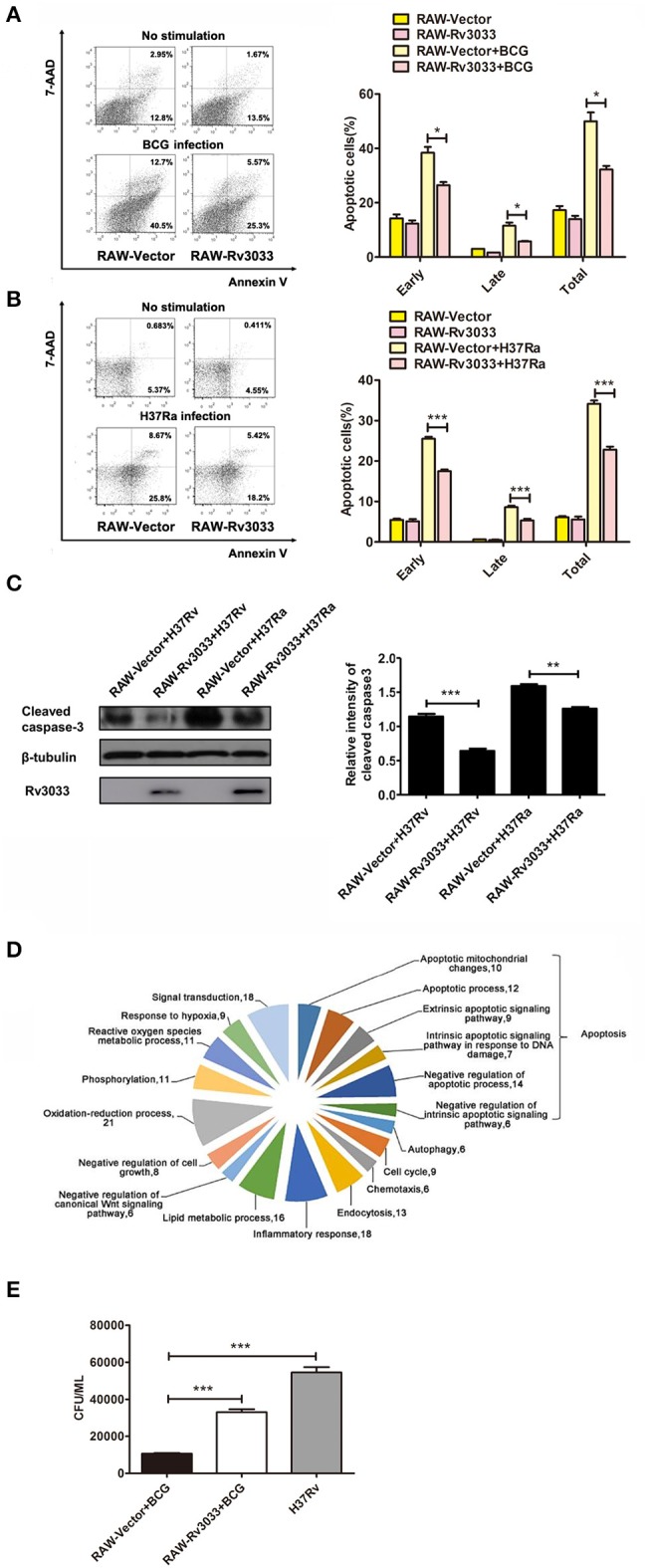
Rv3033 decreased mycobacteria-induced macrophage apoptosis and promoted bacillary survival. **(A)** RAW-Vector and RAW-Rv3033 cells were infected with BCG (MOI = 10) for 36 h. Apoptotic cells were determined by Annexin V/7-AAD staining assays. The percentages of early, late, and total apoptotic cells in FACS assay. **(B)** RAW-Vector and RAW-Rv3033 cells were infected with H37Ra (MOI = 10) for 36 h. Apoptotic cells were determined by Annexin V/7-AAD staining assays. The percentages of early, late, and total apoptotic cells in FACS assay. **(C)** RAW-Vector and RAW-Rv3033 cells were infected with H37Ra or H37Rv (MOI = 10) for 24 h, cleaved caspase3 was detected by western blot. Semi-quantification of cleaved caspase-3. **(D)** RAW-Vector and RAW-Rv3033 cells were infected with BCG (MOI = 10) for 12 h, differentially expressed gene analysis according to biological processes in RAW-Rv3033 compared to RAW-Vector cells. **(E)** CFU counting of intracellular viable bacteria in RAW-Rv3033 with BCG and RAW-Vector cells infected with BCG or H37Rv (MOI = 10) for 2 days. Data are performed as the mean (±SEM) and from one representative experiment of three independent experiments. (^*^p < 0.05; ^**^p < 0.01; ^***^p < 0.001).

To further confirm the Rv3033 anti-apoptosis effects, RAW264.7 cells stably over-expressing Rv3033 (RAW-Rv3033) were established. Following infected with BCG, the apoptotic cell percentages were assessed by Annexin V /7-AAD staining assay. As shown in Figure 3A, compared with the control group (RAW-Vector), infected RAW-Rv3033 cells showed obvious lower apoptotic cell percentages (50 vs. 30%), of note, both the early and late apoptotic cell percentages obviously decreased, indicating that the Rv3033-mediated anti-apoptosis effect occurs at the early stage. This decreased apoptosis was also detected in H37Ra-infected RAW-Rv3033 cells (Figure 3B). And active caspase-3 was significantly decreased in RAW-Rv3033 cells compared with RAW-Vector cells post H37Ra and H37Rv infection (Figure 3C). In addition, these data were further supported by the high-throughput next generation sequencing data, in which Rv3033 overexpression resulted in the alteration of multiple apoptosis-associated gene expression (Figure 3D). Accordingly, RAW-Rv3033 cells had significantly increased bacillary burdens compared to RAW-Vector cells (Figure 3E).

### Rv3033 suppressed mycobacteria-infected macrophage apoptosis by inhibiting the intrinsic but not extrinsic apoptotic pathway

**Figure 4:**
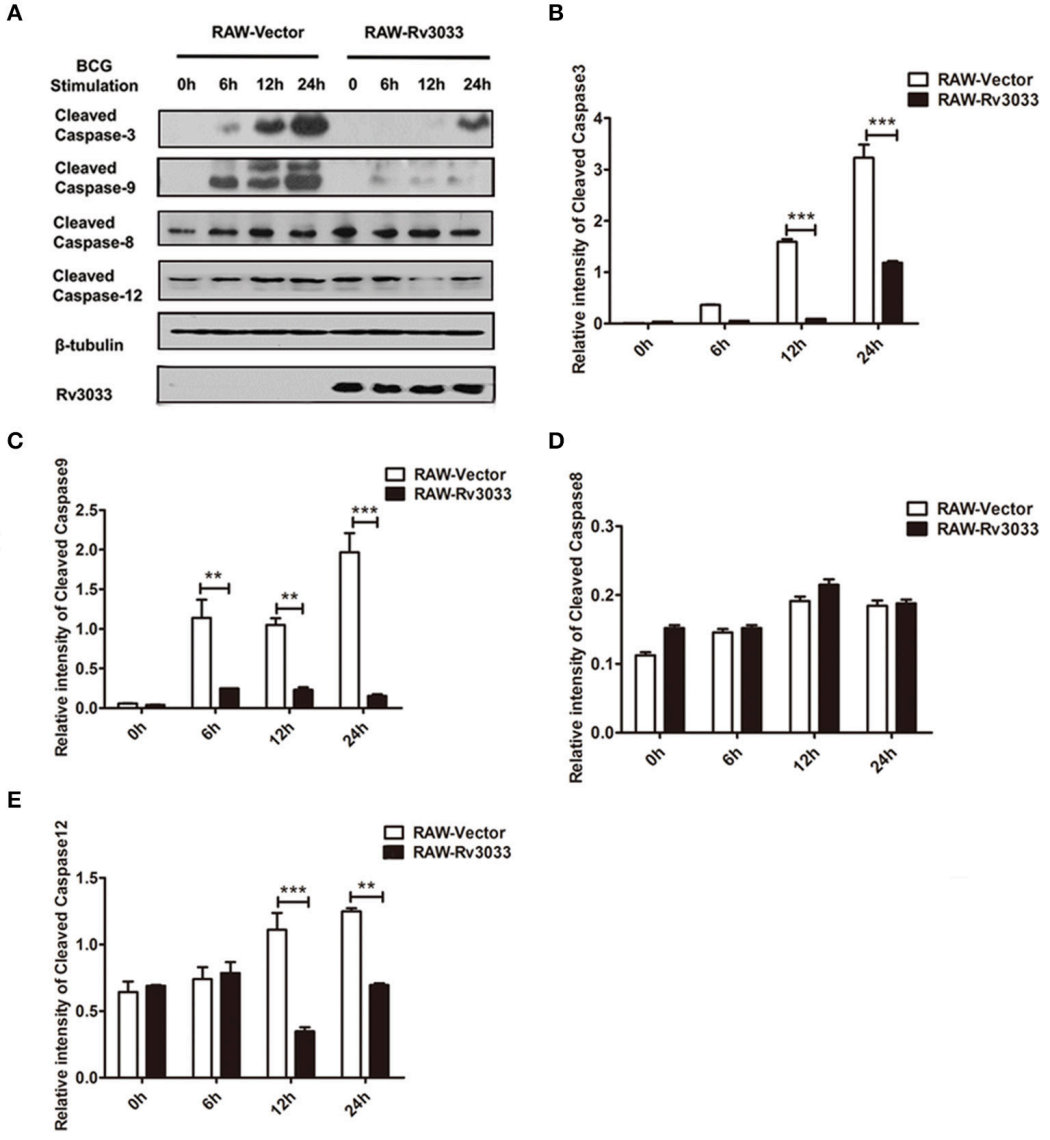
Rv3033 suppressed mycobacteria-induced macrophage apoptosis by inhibiting the intrinsic apoptotic, but not the extrinsic apoptotic pathway. **(A)** RAW-Vector and RAW-Rv3033 cells were infected with BCG (MOI = 10) for 6, 12, or 24 h. Cleaved caspase-3, cleaved caspase-9, cleaved caspase-8, and cleaved caspase-12 protein levels were determined by western blot analyses. **(B–E)** Semi-quantification of cleaved caspase-3, cleaved caspase-9, cleaved caspase-8, and cleaved caspase-12. Data are performed as the mean (±SEM) and from one representative experiment of three independent experiments. (^**^p < 0.01; ^***^p < 0.001).

Apoptosis could be caused by two main pathways: caspase-9-mediated intrinsic pathway and caspase-8-mediated extrinsic pathway, both of which have been reported to be activated post mycobacteria infection (7). These two pathways finally converge on caspase-3 activation and ultimately lead to apoptotic DNA fragmentation via cleaving several target proteins. Herein, we found that active caspase-3 was significantly decreased in RAW-Rv3033 cells compared with RAW-Vector cells, indicative of a lower apoptosis level, which was consistent with our FACS data (Figures 4A,B, 3A). When detected the activation of upstream apoptosis pathways, we found that intrinsic apoptosis molecules (both cleaved caspase-9 and cleaved caspase-12) were obviously reduced in RAW-Rv3033 cells, while extrinsic apoptotic factor caspase-8 activation was not affected (Figures 4C–E), indicating that Rv3033 inhibited the intrinsic but not the extrinsic apoptotic pathway.

### Rv3033 inhibition on intrinsic apoptosis was mediated by blocking the mitochondrial as well as ER stress PERK branch pathway

**Figure 5:**
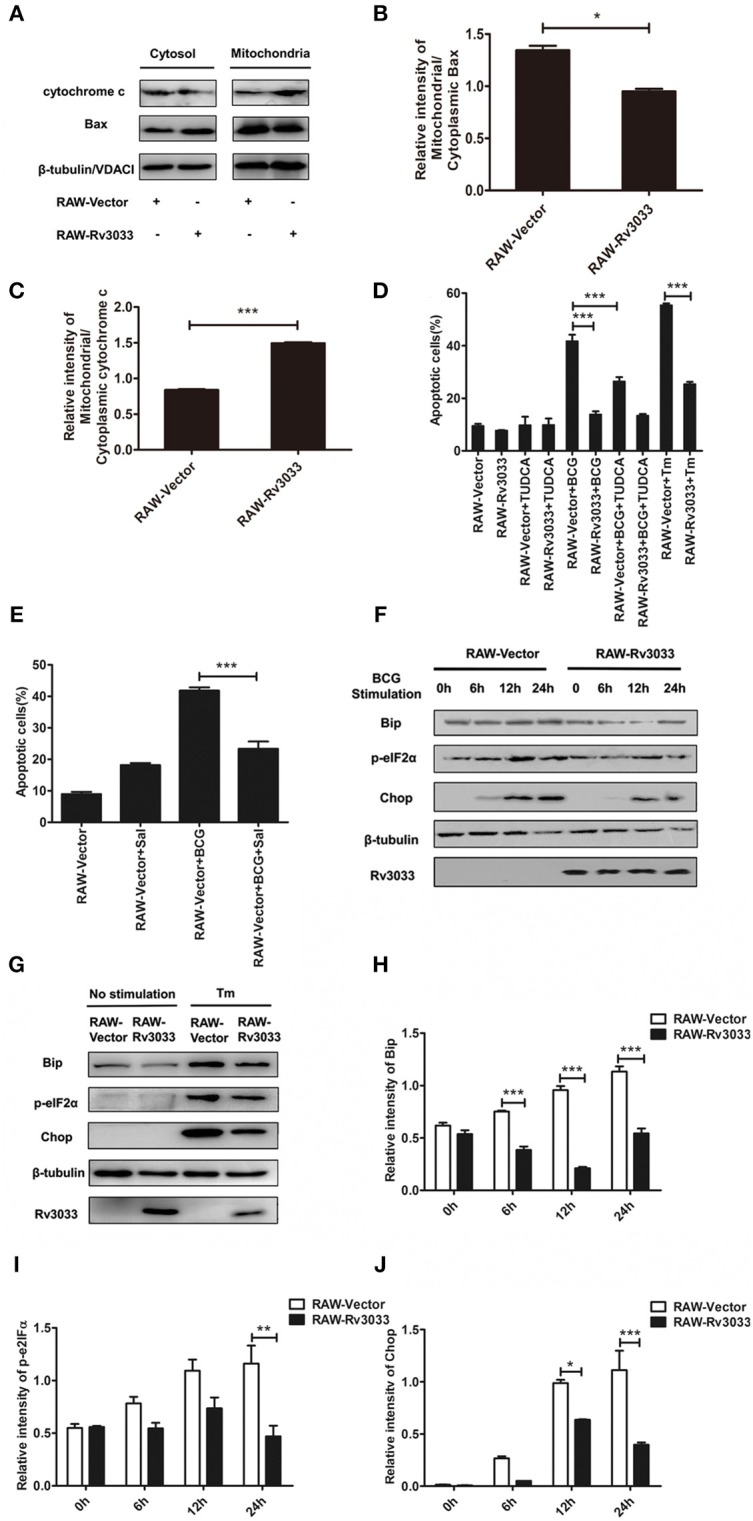
Rv3033 inhibition on intrinsic apoptosis were mediated by blocking the mitochondrial as well as ER stress PERK branch pathways. **(A)** RAW-Vector and RAW-Rv3033 cells were infected with BCG (MOI = 10) for 24 h. Mitochondrial fractions were isolated from whole cell lysates in RAW264.7 cells. The protein levels of Bax and cytochrome c were detected by western blot of the supernatant and mitochondrial fractions. **(B,C)** Semi-quantification of Bax and cytochrome c. **(D)** RAW-Vector and RAW-Rv3033 cells were infected with BCG (MOI = 10) pretreated with or without TUDCA (500 μg/ml) for 36 h. Apoptotic cells were determined by Annexin V/7-AAD staining assays. **(E)** RAW-Vector and RAW-Rv3033 cells were infected with BCG (MOI = 10) pretreated with or without salubrinal (10 μM) for 36 h. Apoptotic cells were determined by Annexin V/7-AAD staining assays. **(F)** RAW-Vector and RAW-Rv3033 cells were infected with BCG (MOI = 10) for 6, 12, or 24 h. Bip, p-eIF2α and chop were determined by western blot analyses. **(G)** RAW-Vector and RAW-Rv3033 cells were stimulated with Tm (5 μg/ml) for 12 h, and Bip, p-eIF2α and Chop were determined by western blot analyses. **(H–J)** Semi-quantification of Bip, p-eIF2α, and Chop. Data are performed as the mean (±SEM) and from one representative experiment of three independent experiments. (^*^p < 0.05; ^**^p < 0.01; ^***^p < 0.001).

Cytochrome c release from mitochondria to cytosol is a canonical trigger for intrinsic apoptosis. To verify whether Rv3033 affected mitochondrial-mediated intrinsic apoptosis after mycobacteria infection, we tested the translocation of the mitochondrial apoptosis-related markers Bax and cytochrome c from the mitochondria to cytosol. As shown in Figures 5A,B, more obvious accumulation cytoplasm Bax were seen in mycobacteria-infected RAW-Rv3033 cells compared to control cells, indicating that Rv3033 inhibited Bax entering into the mitochondria. Consistently, obvious lower cytochrome c translocation from mitochondria to cytosol were observed in mycobacteria-infected RAW-Rv3033 cells compared to control cells, suggesting a lower apoptosis-inducing status of the mitochondria (Figure 5C). These data demonstrated that Rv3033 could prevent mitochondrial-mediated intrinsic apoptosis in mycobacteria-infected macrophages.

Several studies have indicated that intrinsic apoptosis could also be mediated by ER stress after mycobacteria infection (21, 24–27). Therefore, we investigated whether Rv3033 affected ER stress-mediated apoptosis. As shown in Figure 5D, ER stress inhibition by tauroursodeoxycholic acid (TUDCA, a bile acid acting as a potent chemical chaperone that inhibited ER stress) treatment could robustly decrease the RAW-Vector apoptosis (50–30%), indicating that ER stress was involved in mycobacteria-induced macrophage apoptosis. While this phenomenon was absent in RAW-Rv3033 cells. In support of these data, no obvious altered apoptosis were occurred when RAW-Rv3033 were treated with PERK pathway inhibitor (salubrinal) neither (Figure 5E), indicating that ER stress, especially PERK branch-mediated apoptosis was inhibited in Rv3033-over-expressing macrophages compared with the control cells following mycobacteria infection. In consistent with it, we also detected the lower expression of two critical factors of the PERK pathway (p-eIF2α and Chop) both in mycobacteria infection and tunicamycin (Tm, a classical ER stress inducer by inhibiting the synthesis of N-linked glycoproteins)-treated (Figures 5F–J). All of these data showed that Rv3033 could suppress intrinsic apoptosis by simultaneously blocking mitochondrial-mediated and ER stress PERK branch-mediated pathways.

## Discussion

TB is a highly infectious disease that has become a major threat to public health. Currently, although a cocktail of first-line drugs, including isoniazid (INH), rifampin, pyrazinamide (PZA), and ethambutol are effective during the active phase of TB infections (28), the effective treatments for latent infection are still limited which are partly due to the superb immune evasion abilities. In the long-term evolution, mycobacteria adopts several immune evasion strategies to convert macrophages from immune clearance to immune sanctuary (including inhibiting apoptosis, blocking autophagy and modulating inflammation) to facilitate bacillary growth and survival (9). Among them, modulating macrophage apoptosis is an important defense mechanism, but the detailed processes and the corresponding mechanisms remain unclear. To date, exploring and identifying novel potent mycobacterial proteins possessing macrophage apoptosis modulation ability attract more and more attention.

Rv3033, a 19 kDa mycobacterial protein, was first identified by the complete mycobacteria genomic sequencing in 1998 (29), while its function remained unknown until 2005 when suggested as a possible candidate to facilitate mycobacteria survival in macrophages. In this study, we detailedly explored the exact role of Rv3033 in mycobacteria-infected macrophages and tried to decipher the corresponding mechanisms. Firstly, we screened four mycobacterial secreted proteins (Rv0928, Rv1096, Rv3033, and Rv3369). By overexpressing these genes in *M. smegmatis* individually, we found that Rv3033 behaved as the most potent macrophage apoptosis inhibitor and could potently facilitating intracellular mycobacteria survival.

Apoptosis can be induced by extrinsic (caspase-8 mediated) and intrinsic (caspase-9 mediated) pathways, which all play effective roles in eliminating mycobacteria in macrophages (7, 11–13, 30). For example, mycobacteria infection promoted TNF-α production, which induced macrophage extrinsic apoptosis by binding to TNFR1 and limited bacillary survival. Accordingly, mycobacteria have adopted a serial of bacterial proteins to inhibit the macrophage extrinsic apoptosis. Miller et al. reported that mycobacterial virulence factor NADH-ubiquinone oxidoreductase chain G (NuoG) decreased TNF-α-mediated extrinsic apoptosis by neutralizing NOX2-derived ROS production (12). Danelishvili et al. found that mycobacterial Rv3654c suppressed caspase-8-mediated extrinsic apoptosis via binding to protein-associated splicing factor (PSF) in macrophages (18). While, in this study, Rv3033 barely changed the activation extent of the extrinsic apoptosis in mycobacteria-infected macrophages, indicating that it highly possibly fact on the intrinsic apoptosis pathway.

The canonical intrinsic apoptosis is relied on mitochondrial release of pro-apoptotic factor cytochrome c and apoptosis inducing factor (AIF). Following interacting with the apoptotic protease activating factor-1 (Apaf-1), cytochrome c drives the apoptosome assembly and caspase-9 activation, leading to apoptotic DNA fragmentation via cleaving several target proteins (31). To oppose this hostile cellular process, mycobacterial virulence factors, such as PtpA, MPT64, and GroEL2 have been found to inhibit mycobacteria-infected macrophage apoptosis by repressing caspase-9-mediated intrinsic pathway. Poirier et al. (32) showed that mycobacterial PtpA inhibited intrinsic apoptosis by dephosphorylating host GSK3α. Wang et al. (33) found that mycobacterial protein MPT64 repressed macrophage apoptosis through NF-kB-miRNA21-Bcl-2 pathway. Joseph et al. (34) reported that Cpn60.2 (GroEL2) disturbed macrophage mitochondrion-mediated apoptosis via interacting with mortalin. In this study, we found that the Rv3033 over-expression could notably decrease intrinsic apoptosis molecule caspase-9 activation and led to the lower survival of mycobacteria-infected macrophage as well as higher bacterial loads.

Besides of mitochondrial pathway, recently ER stress is also found as a novel apoptosis pathway in mycobacteria-infected macrophages (25, 27, 35). ER stress mainly activates three pathways: IRE1, PERK, and ATF6. Among them, the PERK pathway is the major pathway that induces cell apoptosis and eliminates mycobacteria (26). To date, several experiments have demonstrated that mycobacterial proteins (ESAT-6, 38-kDa, PPE32, HBHA) are involved in ER stress-mediated apoptosis (21–23, 36). Interestingly, all of these virulence factors have been proved to increase ER stress-mediated apoptosis in mycobacteria-infected macrophages. Few mycobacterial protein functions as a repressor of ER stress-mediated apoptosis has been shown. Fortunately, herein we found that unlike the previously identified ER stress apoptosis-promoting protein, virulent Rv3033 protein could potently inhibit the expression of the ER stress PERK branch marker protein Bip/GRP78, p-eIF2α, Chop, and caspase-12, demonstrating that in addition to repressing the canonical (mitochondrial-mediated) intrinsic apoptosis pathway, Rv3033 could also simultaneously suppress the novel ER stress-(PERK-eIF2a-Chop-) mediated intrinsic apoptosis. This further emphasized the important role of Rv3033 in favoring intracellular bacillary survival, as compared with the most identified apoptosis-inhibiting virulence proteins which usually acts on solo apoptosis pathway, Rv3033 could more widely repress intrinsic apoptosis by orchestrating blockade of mitochondrial cytochrome c release and endoplasmic reticulum (ER) stress PERK branch activation.

In conclusion, we identified a novel function of the mycobacterial virulence factor Rv3033 as facilitating latent infection by inhibiting macrophage intrinsic apoptosis. This function relied on simultaneously blockade of both mitochondrial cytochrome c release and endoplasmic reticulum (ER) stress PERK branch activation. This might make Rv3033 as a more promising therapeutic target to control the latent mycobacteria infection.

## Author contributions

SX conceived this study. YY revised the manuscript. YD designed the experiments. WZ performed the experiments, analyzed the results, and drafted the manuscript. QL constructed the Ms::Rv1096 strain and assembled the Figure 2.

### Conflict of interest statement

The authors declare that the research was conducted in the absence of any commercial or financial relationships that could be construed as a potential conflict of interest.

## References

[1] WHO (2017). Global Tuberculisis Report. WHO.

[2] Hmama Z Pena-Diaz S Joseph S Av-Gay Y. Immunoevasion and immunosuppression of the macrophage by Mycobacterium tuberculosis. Immunol Rev. (2015) 264:220–32. 10.1111/imr.12268 25703562

[3] Berrington WR Hawn TR. Mycobacterium tuberculosis, macrophages, and the innate immune response: does common variation matter? Immunol Rev. (2007) 219:167–86. 10.1111/j.1600-065X.2007.00545.x 17850489 PMC2859969

[4] Sia JK Georgieva M Rengarajan J. Innate immune defenses in human tuberculosis: an overview of the interactions between *Mycobacterium tuberculosis* and innate immune cells. J Immunol Res. (2015) 2015:747543. 10.1155/2015/747543 26258152 PMC4516846

[5] Weiss G Schaible UE. Macrophage defense mechanisms against intracellular bacteria. Immunol Rev. (2015) 264:182–203. 10.1111/imr.12266 25703560 PMC4368383

[6] Bermudez LE Danelishvili L Babrack L Pham T. Evidence for genes associated with the ability of *Mycobacterium avium* subsp. hominissuis to escape apoptotic macrophages. Front Cell Infect Microbiol. (2015) 5:63. 10.3389/fcimb.2015.00063 26380226 PMC4548235

[7] Behar SM Martin CJ Booty MG Nishimura T Zhao X Gan HX. Apoptosis is an innate defense function of macrophages against Mycobacterium tuberculosis. Mucosal Immunol. (2011) 4:279–87. 10.1038/mi.2011.3 21307848 PMC3155700

[8] Lam A Prabhu R Gross CM Riesenberg LA Singh V Aggarwal S. Role of apoptosis and autophagy in tuberculosis. Am J Physiol Lung Cell Mol Physiol. (2017) 313:L218–29. 10.1152/ajplung.00162.2017 28495854 PMC5582934

[9] Liu CH Liu H Ge B. Innate immunity in tuberculosis: host defense vs pathogen evasion. Cell Mol Immunol. (2017) 14:963–75. 10.1038/cmi.2017.88 28890547 PMC5719146

[10] Edwards KM Cynamon MH Voladri RK Hager CC DeStefano MS Tham KT. Iron-cofactored superoxide dismutase inhibits host responses to Mycobacterium tuberculosis. Am J Respir Crit Care Med. (2001) 164:2213–9. 10.1164/ajrccm.164.12.2106093 11751190

[11] Velmurugan K Chen B Miller JL Azogue S Gurses S Hsu T. *Mycobacterium tuberculosis* nuoG is a virulence gene that inhibits apoptosis of infected host cells. PLoS Pathog. (2007) 3:e110. 10.1371/journal.ppat.0030110 17658950 PMC1924871

[12] Miller JL Velmurugan K Cowan MJ Briken V. The type I NADH dehydrogenase of *Mycobacterium tuberculosis* counters phagosomal NOX2 activity to inhibit TNF-alpha-mediated host cell apoptosis. PLoS Pathog. (2010) 6:e1000864. 10.1371/journal.ppat.1000864 20421951 PMC2858756

[13] Shin DM Jeon BY Lee HM Jin HS Yuk JM Song CH. *Mycobacterium tuberculosis* eis regulates autophagy, inflammation, and cell death through redox-dependent signaling. PLoS Pathog. (2010) 6:e1001230. 10.1371/journal.ppat.1001230 21187903 PMC3002989

[14] Danelishvili L Everman JL McNamara MJ Bermudez LE. Inhibition of the Plasma-Membrane-Associated Serine Protease Cathepsin G by *Mycobacterium tuberculosis* Rv3364c Suppresses Caspase-1 and Pyroptosis in Macrophages. Front Microbiol. (2011) 2:281. 10.3389/fmicb.2011.00281 22275911 PMC3257866

[15] Rengarajan J Bloom BR Rubin EJ. Genome-wide requirements for *Mycobacterium tuberculosis* adaptation and survival in macrophages. Proc Natl Acad Sci USA. (2005) 102:8327–32. 10.1073/pnas.0503272102 15928073 PMC1142121

[16] Bauerfeld CP Rastogi R Pirockinaite G Lee I Huttemann M Monks B. TLR4-mediated AKT activation is MyD88/TRIF dependent and critical for induction of oxidative phosphorylation and mitochondrial transcription factor A in murine macrophages. J Immunol. (2012) 188:2847–57. 10.4049/jimmunol.1102157 22312125 PMC3294201

[17] Jayakumar D Jacobs WR Jr Narayanan S. Protein kinase E of *Mycobacterium tuberculosis* has a role in the nitric oxide stress response and apoptosis in a human macrophage model of infection. Cell Microbiol. (2008) 10:365–74. 10.1111/j.1462-5822.2007.01049.x 17892498

[18] Danelishvili L Yamazaki Y Selker J Bermudez LE. Secreted *Mycobacterium tuberculosis* Rv3654c and Rv3655c proteins participate in the suppression of macrophage apoptosis. PLoS ONE (2010) 5:e10474. 10.1371/journal.pone.0010474 20454556 PMC2864267

[19] Guo S Xue R Li Y Wang SM Ren L Xu JJ. The CFP10/ESAT6 complex of *Mycobacterium tuberculosis* may function as a regulator of macrophage cell death at different stages of tuberculosis infection. Med Hypotheses (2012) 78:389–92. 10.1016/j.mehy.2011.11.022 22192908

[20] Briken V. *Mycobacterium tuberculosis* genes involved in regulation of host cell death. Adv Exp Med Biol. (2013) 783:93–102. 10.1007/978-1-4614-6111-1_5 23468105

[21] Choi JA Lim YJ Cho SN Lee JH Jeong JA Kim EJ. Mycobacterial HBHA induces endoplasmic reticulum stress-mediated apoptosis through the generation of reactive oxygen species and cytosolic Ca2+ in murine macrophage RAW 264.7 cells. Cell Death Dis. (2013) 4:e957. 10.1038/cddis.2013.489 24336077 PMC3877560

[22] Lim YJ Choi JA Lee JH Choi CH Kim HJ Song CH. *Mycobacterium tuberculosis* 38-kDa antigen induces endoplasmic reticulum stress-mediated apoptosis via toll-like receptor 2/4. Apoptosis (2015) 20:358–370. 10.1007/s10495-014-1080-2 25544271

[23] Deng W Yang W Zeng J Abdalla AE Xie J. *Mycobacterium tuberculosis* PPE32 promotes cytokines production and host cell apoptosis through caspase cascade accompanying with enhanced ER stress response. Oncotarget (2016) 7:67347–59. 10.18632/oncotarget.12030 27634911 PMC5341880

[24] Seimon TA Kim MJ Blumenthal A Koo J Ehrt S Wainwright H. Induction of ER stress in macrophages of tuberculosis granulomas. PLoS ONE (2010) 5:e12772. 10.1371/journal.pone.0012772 20856677 PMC2939897

[25] Lim YJ Choi JA Choi HH Cho SN Kim HJ Jo EK. Endoplasmic reticulum stress pathway-mediated apoptosis in macrophages contributes to the survival of Mycobacterium tuberculosis. PLoS ONE (2011) 6:e28531. 10.1371/journal.pone.0028531 22194844 PMC3237454

[26] Cui Y Zhao D Barrow PA Zhou X. The endoplasmic reticulum stress response: A link with tuberculosis? Tuberculosis (Edinb) (2016) 97:52–6. 10.1016/j.tube.2015.12.009 26980496

[27] Cui Y Zhao D Sreevatsan S Liu C Yang W Song Z. *Mycobacterium bovis* induces endoplasmic reticulum stress mediated-apoptosis by activating IRF3 in a murine macrophage cell line. Front Cell Infect Microbiol. (2016) 6:182. 10.3389/fcimb.2016.00182 28018864 PMC5149527

[28] Kim JJ Lee HM Shin DM Kim W Yuk JM Jin HS. Host cell autophagy activated by antibiotics is required for their effective antimycobacterial drug action. Cell Host Microbe (2012) 11:457–468. 10.1016/j.chom.2012.03.008 22607799

[29] Cole ST Brosch R Parkhill J Garnier T Churcher C Harris D. Deciphering the biology of *Mycobacterium tuberculosis* from the complete genome sequence. Nature (1998) 393:537–44. 10.1038/31159 9634230

[30] Zhang J Jiang R Takayama H Tanaka Y. Survival of virulent *Mycobacterium tuberculosis* involves preventing apoptosis induced by Bcl-2 upregulation and release resulting from necrosis in J774 macrophages. Microbiol Immunol. (2005) 49:845–52. 10.1111/j.1348-0421.2005.tb03673.x 16172539

[31] Parandhaman DK Narayanan S. Cell death paradigms in the pathogenesis of *Mycobacterium tuberculosis* infection. Front Cell Infect Microbiol. (2014) 4:31. 10.3389/fcimb.2014.00031 24634891 PMC3943388

[32] Poirier V Bach H Av-Gay Y. *Mycobacterium tuberculosis* promotes anti-apoptotic activity of the macrophage by PtpA protein-dependent dephosphorylation of host GSK3alpha. J Biol Chem. (2014) 289:29376–85. 10.1074/jbc.M114.582502 25187516 PMC4200286

[33] Wang Q Liu S Tang Y Liu Q Yao Y. MPT64 protein from *Mycobacterium tuberculosis* inhibits apoptosis of macrophages through NF-kB-miRNA21-Bcl-2 pathway. PLoS ONE (2014) 9:e100949. 10.1371/journal.pone.0100949 25000291 PMC4085073

[34] Joseph S Yuen A Singh V Hmama Z. *Mycobacterium tuberculosis* Cpn60.2 (GroEL2) blocks macrophage apoptosis via interaction with mitochondrial mortalin. Biol Open (2017) 6:481–8. 10.1242/bio.023119 28288970 PMC5399554

[35] Lim YJ Choi HH Choi JA Jeong JA Cho SN Lee JH Mycobacterium kansasii-induced death of murine macrophages involves endoplasmic reticulum stress responses mediated by reactive oxygen species generation or calpain activation. Apoptosis (2013) 18:150–9. 10.1007/s10495-012-0792-4 23264129

[36] Choi HH Shin DM Kang G Kim KH Park JB Hur GM. Endoplasmic reticulum stress response is involved in *Mycobacterium tuberculosis* protein ESAT-6-mediated apoptosis. FEBS Lett. (2010) 584:2445–54. 10.1016/j.febslet.2010.04.050 20416295

